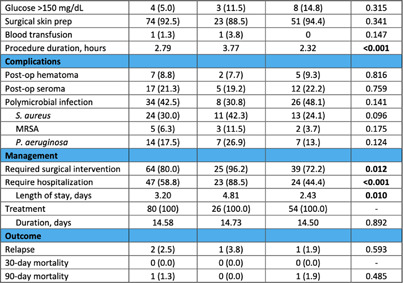# Title: Risk Factors Associated with Breast Surgical Site Infections in Patient with Implants in Southeast Michigan

**DOI:** 10.1017/ash.2025.411

**Published:** 2025-09-24

**Authors:** Simran Brar, Geehan Suleyman

**Affiliations:** 1 Henry Ford Health; 2 Henry Ford Health

## Abstract

**Background:** Surgical site infections (SSIs) are one of the most common complications of breast surgery and a major source of postoperative morbidity and mortality. The risk of developing breast SSI (BSSI) is further heightened by the insertion of implants during breast surgery. The objective of this study is to describe clinical outcomes in patients with breast SSI and to compare patients with implants to those without. **Methods:** Retrospective case series of adult patients with clinical BSSI at Henry Ford Health, an integrated health care organization that includes 5 hospitals and 9 emergency departments (EDs) in Southeast Michigan, from January 2021 to December 2023. Cases that met National Healthcare Safety Network (NHSN) BSSI criteria were screened for clinical infection. Demographics, comorbidities, risk factors, microbiological data, and clinical outcomes were included. **Results:** Eighty (54%) of 147 cases that met NHSN criteria had clinical infection, and 26 (32.5%) had implants placed (Table). Most patients were female (95%) and white (63%) with a mean age of 51.7 years and BMI >30 (35%). The majority had active breast cancer (56%) and underwent biopsy within 1 year (66%), which were significantly more common in the implant group (81% vs. 44%, p=0.002, and 85% vs 57%, p=0.002, respectively). Malignancy (55%) was the primary indication for the surgery; mastectomy with or without lymph node dissection was the most frequently performed procedure in both groups. The implant group had a significantly longer procedure duration (3.77 vs. 2.32 hours, p < 0 .001). Post-operative complications did not differ significantly among the two groups. The implant group was more likely to require hospitalization (88% vs. 44%, p < 0 .001) with a longer length of stay and surgical intervention (96% vs. 72%, p=0.012). All patients were treated with antibiotics, and outcomes were similar between the two groups. **Conclusion:** In this cohort of patients with BSSI, the presence of breast implants was associated with a significantly higher risk of hospitalization and surgical intervention. Identifying risk factors in these patients is essential to reduce the need for hospitalization and additional surgical procedures.

**Figure:**
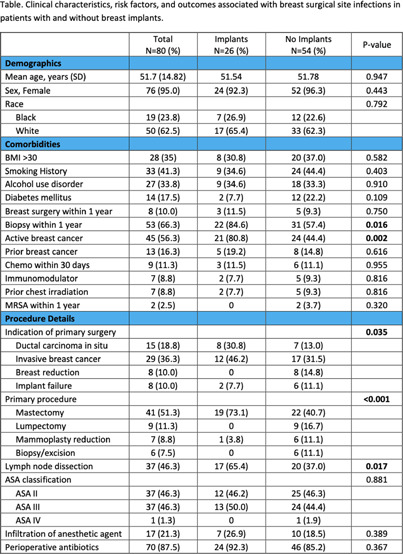

**Figure:**